# Epinephrine auto-injector needle length: The impact of winter clothing

**DOI:** 10.1186/s13223-020-00422-4

**Published:** 2020-04-15

**Authors:** Sten Dreborg, Gina Tsai, Harold Kim

**Affiliations:** 1 Department Child and Adolescent Allergology, Women’s and Children’s Health, University of Uppsala, Uppsala, Sweden; 2 Department of Medicine, Western University, London, Canada; 3 Department of Medicine, McMaster University, Hamilton, Canada

**Keywords:** Auto-injector, Epinephrine, Intramuscular, Subcutaneous, Intraosseous, Periosteal, Skin to bone distance, Skin to muscle distance, Clothing

## Abstract

**Background:**

Epinephrine auto-injectors are expected to deliver the drug intramuscularly.

**Objective:**

To study whether injection through clothing influences the frequency of subcutaneous and intraosseous/periosteal deposition of epinephrine.

**Methods:**

Skin to muscle and skin to bone distances were measured for 303 children and adolescents and 99 adults. Distance was determined by ultrasound, with high or low pressure on the ultrasound probe. The risk/percentage of subcutaneous and intraosseous/periosteal injections was calculated using the lower and upper limits for the authority-approved length of EAI needles as provided by two high pressure EAI manufacturers and one low pressure EAI manufacturer. The addition winter clothing on the delivery of epinephrine was illustrated by comparing drug delivery fissue depth with no clothes. Furthermore, the riof non-intramuscular delivery for the shortest and longest approved needle length was calculated.

**Results:**

When using EpipenJr^®^ in children < 15 kg the risk of intraosseous/periostal injection was reduced from 1% and 59% for the shortest and longest approved needle length to 0 and 15% with winter clothes. The Auvi-Q^®^ 0.1 mg had no risk of intraosseous/periosteal injection. However, the subcutaneous deposition risk increased from 94% and 28% to 100% and 99% with winter clothes. The risk of subcutaneous injection using EpipenJr^®^ in the youngest children increased from 13% and 0% to 81% and 1% with winter clothes, and with Epipen^®^ in adults from 45% and 17% to 60% and 38%. Emerade^®^, had a risk of subcutaneous injection in adults increasing from 14% and 10% to 28% and 21% adding winter clothes.

**Conclusion:**

The risk of intraosseous/periosteal injections decreases and the risk of subcutaneous injection increases when injecting through winter clothes for all EAIs.

## Background

Epinephrine is indicated for intramuscular injection in the treatment of anaphylaxis [1]. We recently reported on the risk for subcutaneous and intraosseous/periosteal injections, using ultrasound for measuring the distance from skin to muscle and bone, respectively [2–6]. There was a risk of subcutaneous injection when using both high pressure EAIs (HPEAIs) with 94% risk in small children using Auvi-Q^®^ 0.1 mg and in about 28% in adult overweight women using Epipen^®^ [2]. In addition, there was a 71% risk risk of an intraosseous/periosteal injection in small children when using Auvi-Q^®^ 0.15 mg HPEAIs [5].

There are two important factors influencing the risk of intraosseous/periosteal injection and subcutaneous injection. The pressure applied and the length of the needle penetrating the skin.

We applied low pressure on the ultrasound probe to mimic the pressure applied on low pressure EAIs (LPEAIs) and higher pressure (about 8 lb or 35 Newtons (N)) on the probe to mimic the pressure applied to HPEAIs [2–4].

Our data were based on ultrasound estimation of the naked skin to muscle distance and the naked skin to bone distance, respectively. In clinical practice, allergists typically suggest that these devices can and should be delivered through clothing. In colder climates, people often wear thicker clothing during the winter months. There is no published data on the possible influence of thick clothing on skin to muscle distance and skin to bone distance using HPEAIs or LPEAIs. This information may be clinically helpful in predicting expected intramuscular delivery of epinephrine.

The aim of this communication is to study the influence of winter clothing on the risk for intraosseous/periosteal and subcutaneous injection with currently available EAIs, taking in account the variation in needle length within batches of EAIs released for marketing.

## Methods

Four hundred and one (401) consecutive patients with diagnosed food allergy were included. As described earlier, 302 children and adolescents and 99 adults (67 women) underwent ultrasound investigations using high (8 lb = 35 N) and minimal pressure on the probe, noting the skin to bone distance and skin to muscle distance on the mid third of the anterio-lateral aspect of the right thigh [2–4]. Clinical data and basic statistical analyses have been published [2–4]. Moreover, the possible risk of having a subcutaneous instead of an intramuscular injection and the possible risk of having an intraosseous/periosteal injection was analysed in two previous papers [5, 6]. The main findings were an increased risk of subcutaneous injection in adolescents and especially overweight adult women [2, 5]. Furthermore, EAI needles of the same brand vary in length. The shortest allowable needles increase the risk of subcutaneous injection, the longest the risk of intraosseous/periosteal injection [6].

There were two groups of children less than 12 years of age: 0–15 kg (n = 100) [3], 15–30 kg (n = 102) and one group of adolescents > 12 years of age and weighing more than 30 kg (n = 100) [4], totalling 302 (125 girls and 177 boys). Furthermore, 99 adults (18–72 yrs, 67 females), were included in the study [2]. Patients, parents or legal guardians provided written, informed consent before participating in the original studies [2–4].

The needle full length when the EAI is applied on naked skin, and through thick winter cloth was studied. To illustrate the risks, we used the maximum length and the minimum length passing internal controls, Table 1, as earlier described [6].

**Table 1:** Auto-injector needles available in North America and Europe in 2019

EAI	Lower and upper limits for needle length	Naked skin		With thick clothes 3 mm		Pressure against the thigh
		Skin to muscle distance −2 mm (acc. to Diacono)	Skin to bone distance full length	Skin to muscle distance (-3 mm clothes and −2 mm) = − 5 mm	Skin to bone distance full pene-trating needle length − 3 mm	
HPEAI^a^						
Epipen Jr^®^ 0.15 mg	Lower limit	8	10	5	7	Press hard
	Upper limit	13	15	10	12	
Epipen^®^ 0.3 mg	Lower limit	11	13	8	10	
	Upper limit	16	18	13	15	
Auvi-Q 0.1 mm	Lower limit	4.4	6.4	1.4	3.4	Push firmly
	Upper limit	6.9	8.9	3.9	5.9	
Auvi-Q 0.15 mg	Lower limit	9.4	11.4	6.4	8.4	
	Upper limit	12	14.0	9	11	
Auvi-Q 0.3 mg	Lower limit	12.7	14.7	9.7	11.7	
	Upper limit	15.3	17.3	12.3	14.3	
LPEAI^b^						
Emerade^®^ 0.15 mg	Lower limit	13	15	10.0	12.0	Slight pressure
	Upper limit	14.7	16.7	11.7	13.7	
Emerade^®^ 0.3 mg	Lower limit	20.1	22.1	17.1	19.1	
Slight preesure	Upper limit	21.6	23.6	18.6	20.6	
Emerade^®^ 0.5 mg	Lower limit	20.1	22.1	17.1	19.1	
	Upper limit	21.6	23.6	18.6	20.6	

Needle lengths are given according to the manufacturers’ approved specifications. The skin to muscle distance is based on Diacono et al. [7] by subtracting 2 mm from the penetrating needle length. The increased distance to muscle, *i.e.* 2 mm for the eye of the needle. The skin to bone distance is based on the full length of the needle. Both skin to muscle distance and skin to bone distance are given for the case injection is performed on naked skin and with winter cloths. The thickness of winter clothes is proposed to be 3 mm, but can vary among individuals

^a^HPEAI, These devices are high-pressure epinephrine autoinjectors, HPEAIs

^b^This device is a low-pressure epinephrine autoinjector, LPEAI

Recently, Diacono et al. [7] found the whole needle orifice must pass completely into the muscle for proper administration of an intramuscular injection. The skin to muscle distance is measured from the skin surface to the outer side of the fascia. The needle must pass through the fascia and the epimysium into the muscle. If part of the orifice of the needle is within the epimysium during the injection, epinephrine may spread within the loose epimysium tissue. The length of the needle’s eye of the EAIs was estimated to be 2 mm [5]. Therefore, the needle length was reduced by 2 mm when estimating the risk for subcutaneous injection. The thickness of pants worn by children, adolescents and adults varies much. We measured the thickness of 3 winter pants. And with compression, the thickest of them was about three millimeters. The three mm is just an example, it illustrates that the thickness of clothing influences the outcome of injection with EAIs. Therefore, the needle lengths were reduced by 3 mm when estimating the risks for intraosseous/periosteal or subcutaneous injection when wearing thick winter clothes. In case of much thicker or thinner clothes, approximate risks can be calculated from the figures. The needle lengths used for calculation of the risk of intraosseous/periosteal and subcutaneous injection are given in Table 1.

## Outcome variables

We used two primary outcome variables: the proportion of children with (1) skin to bone distance less than the total needle length and that length minus 3 mm (thick clothing) and (2) skin to muscle distance more than the needle length minus 2 mm, and the needle length minus 5 mm (Table 1). Furthermore, the key evaluation parameter is the change of the risk of subcutaneous and intraosseous/periosteal injection when injecting through thick winter clothes. In the results section, we give the percent at risk using the shortest needle%—the longest needle%.

## Statistics

Basic statistical significances and correlations have been reported previously [5–7].

We estimated the proportion of subjects who would likely receive epinephrine intraosseous/periosteal or subcutaneous, respectively, using high and low pressure EAIs.

Since differences in distance are small and therefore data approximate, we have proposed the use of risk classes rather than exact data [6]. The color codes indicating classes of risk are:

- white color, indicates very low risk, 0–2%,
- green color, indicates low risk, 3%–9 %,
- orange color indicates medium risk, 10%–19% and
- red color indicates high risk, *i.e.* higher risk than 20%, for intraosseous/periosteal injection. See also outcome parameters and Table 2.

**Table 2:**
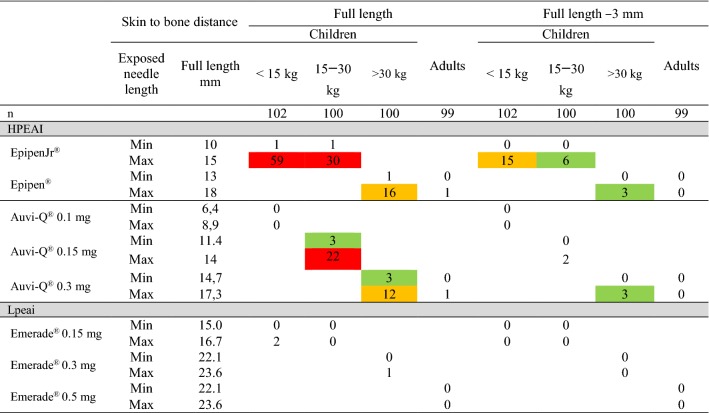
The skin to bone distance in relation to weight

The full length and the full length minus 3 mm, the proposed penetrating needle length wearing winter clothes, was used at calculation of skin to bone distance. The risk of intraosseous/periosteal injection was calculated for both the longest and the shortest needle passing quality control. The weight limits were (< 15 kg), 15–30 kg, adolescents weighing > 30 kg and adults, Table 1

White color indicates very low risk, 0%–2%, green color indicates low risk, 3%–9 %, orange color indicates medium risk, 10%–19%, red color higher risk than 20%, for intraosseous/periosteal injection. The exposed needle lengths are given in mm

## Results

### Patient sample

The basic results of the samples investigated have been published separately elsewhere [5–7]. In this study, we tested whether wearing thick clothes would effect the deposition of epinephrine, and taking also in account the variation in needle length, *i.e.* if using the shortest and the longest needle passing the quality control of the currently available EAIs, would affect the risk of subcutaneous injections or intraosseous/periosteal injections.

We tested one thickness of clothes, i.e. 3 mm, compared to naked skin.

Since the EAI brands had different limits for acceptance of minimum and maximum length of the needle, the impact of variation in length has been analysed for each brand of EAI, Table 1.

### Skin to bone distance

**Fig. 1:**
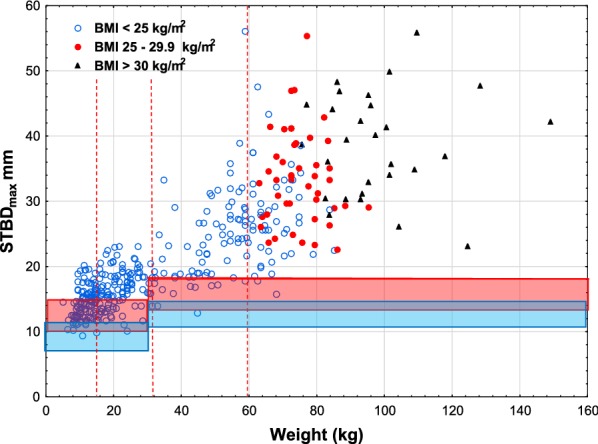
Skin to bone distance _max_, versus weight using Epipen^®^/EpipenJr^®^ EAIs. The full exposed length of the needles with upper and lower limits (red lines) with the variation indicated by the red area and the full length minus 3 mm for winter clothing shown below (blue lines and area). The vertical lines indicate the shift in dose from 0.15 mg to 0.3 mg and from 0.3 mg to 0.5 mg, respectively. BMI limits for adults and symbols are indicated in the left upper corner

**Fig. 2:**
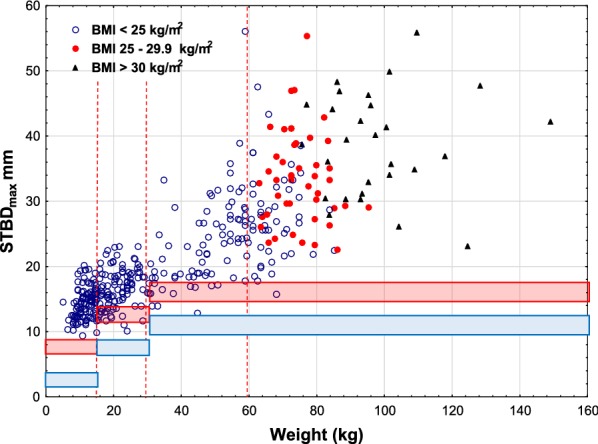
Skin to bone distance _max_, versus weight using Auvi-Q^®^ EAIs. The legend to Fig. 1 explains the lines

The risk for intraosseous/periosteal injection was highest, 1% and 59%, using EpipenJr^®^ in children weighing less than 15 kg, decreasing to 0 and 15% when injected trough thick clothing and that of EpipenJr^®^ 0.15 mg in children weighing 15–30 kg from 1 and 30% to 0 and 6%, Table 2, Figs. 1 and 2.

Using the newly lauched Auvi-Q^®^ 0.1 mg EAI, designed to avoid intraosseous/periosteal injection, showed no risk (0%) of intraosseous/periosteal injection in children less than 15 kg. In children 15–30 kg, the Auvi-Q^®^ 0.15 mg had a 22% and 3% risk of intraosseous/periosteal injection, Table 2, Fig. 2.

The LPEAI Emerade^®^ had very low risk of intraosseous/periosteal penetration in young children.

Winter clothing reduced the risk of intraosseous/periosteal deposition in all age groups.

## Skin to muscle distance

Using the HPEAI Epipen^®^/Epipen Jr^®^, the percentage of children less than 15 kg increased from 13% and 0% to 81% and 1% and in those weighing 15–30 kg rom 8% and 0% to 71% and 1%, when using thick clothes. The gap depending on the difference in needle length allowed for batch release.

When using the new Auvi-Q^®^ 0.10 mg EAI in small children on naked skin, there was a very high risk of subcutaneous injection, 94% and 28%, that was increased when injecting trough winter clothes, to 100% and -99%, Table 3, Fig. 4.

**Table 3:**
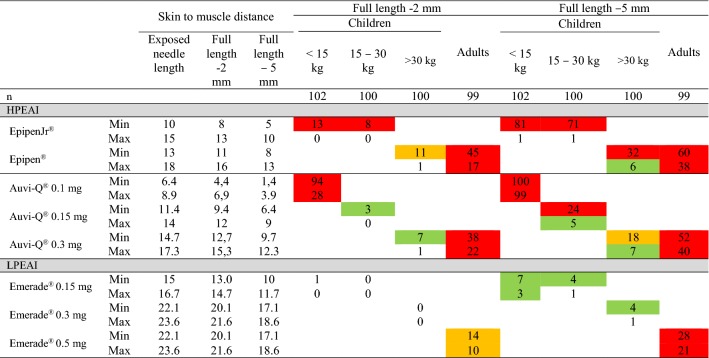
The skin to muscle distance in relation to weigh

Weight limits and color coding as in Table 2. The full length minus 2 mm, due to the finding by Diacono et al. [7] that the full needle eye must pass the endomysium into the muscle and the full length minus 5 mm, *i.e.* 2 mm according to Diacono [7] and the proposed thickness of winter clothes, 3 mm, was used at calculation of skin to muscle distance. The risk of subcutaneous injection was calculated for both the longest and the shortest needle passing quality control

Using Emerade^®^, the risk of subcutaneous injection was intermediate, 14% and 10%, in adults and increased to 28% and 21% when wearing winter clothes, Table 3 and Fig. 5.

Winter clothing increased the risk of subcutaneous injection in all age groups.

## Discussion

In recent years the risk of subcutaneous or intraosseous injection of epinephrine using EAIs has been widely discussed. This paper is based on data from three original publications [2–4]. Those three studies represents the most extensive investigation of the relationships between the distances from skin to muscle, and skin surface to the bone. Distances were determined by ultrasound at the mid anterolateral aspect of the thigh, the recommended area for intramuscular injection of epinephrine using EAIs [8].

The influence of thick clothing on the deposition of epinephrine has not been investigated previously. In this paper, we analysed the influence of thick clothing on skin to muscle distance and skin to bone distance vs. weight, the most commonly used parameter for dosing epinephrine. All winter clothing does not have the same thickness. We decided to use 3 mm and to estimate compressed winter clothing thickness based on caliper measurements. The result of thicker or less thick winter clothing can easily be calculated from the figures in this paper.

The true distance from skin to muscle and bone during the delivery of epinephrine with an EAI will vary with the pressure applied to release the needle of the EAI [2–6]. In previous studies, we identified that EAIs that require high pressure likely compress primarily muscle tissue, which reduces the distance from skin surface to the bone. We estimate that about 90% of the compression originates from compression of the muscle and not from compression of the subcutaneous tissue [2–4].

In a previous paper, we used the limits for acceptance of needle lengths from the manufacturers’ internal specifications, kindly supplied by the manufacturers [6]. This data was also used in this communication. The risk of intraosseous/periosteal penetration was most pronounced using EpipenJr^®^ in small children. The likelihood of subcutaneous injection was highest with the newly introduced Auvi-Q^®^ 0.1 mg epinephrine EAI and in adult obese women [2]. Our findings suggest that it is difficult to obtain reduced risk of both intraosseous/periosteal injection and subcutaneous injection using the same HPEAI. A rough estimate would be to calculate the minimum value of intraosseous/periosteal. Injection combined with the minimum number subcutaneous injection.

In this study, we found the highest risk for intraosseous/periosteal injection at 59 and 1% in children weighing less than 15 kg when using the longest needle of EpipenJr^®^ that is accepted by the manufacturer. Thick clothing reduced the risk to 15 and 0%.

According to our data, the Auvi-Q^®^ 0.1 mg has an estimated risk of bone injection in children less than 15 kg of 0%. However, this EAI has a marked increase in subcutaneous injection from 28 and 94% for naked skin to 100 and 99% if injected through winter clothing, Table 3. This illustrates the difficulty to design an EAI that has both a low risk of intraosseous/periosteal injection and subcutaneous injection.

In adults, winter clothing reduced the risk of intraosseous/periosteal injection from 16 and 3% using the longest needles of Epipen^®^ and from 12 and 3% using Auvi-Q^®^. Emerade^®^ had no risk of intraosseous/periosteal injection in adults.

On the other hand, using the shortest approved needles in adults, the risk of subcutaneous injection increased for Epipen^®^ from 45% to 60%, for Auvi-Q^®^ from 38 to 52% and for Emerade from 14 to 28%.

It would be desirable to have a longer needle length available in EAIs for the obese and overweight adults having the risk of subcutaneous injection. The risk for these patients must be better defined than by weight.

It may be possible to better characterize patients to identify those at risk for subcutaneous injection by Auvi-Q^®^ 0.1 mg, intraosseous/periosteal injection using EpipenJr^®^ and Auvi-Q 0.15 mg, and adults at risk of subcutaneous injection.

In general, winter clothing reduced the risk of intraosseous/periosteal injection in children and increased the risk of subcutaneous injection in adults and in children using Auvi-Q^®^ 0.1 mg epinephrine EAIs.

In this series of studies [2–4], we used 8 lb or about 35 Newtons (N) as high pressure and applied a low pressure to mimic the required pressure to release the needle of HPEAIs and LPEAIs, respectively. The declared variation in pressure that is accepted by companies for release of new batches, applied to EAIs has been presented elsewhere [9]. There are instruments that can apply a specified pressure to the ultrasound probe and such instruments should be used in all future trials and in the instruction to prescribing health care personnel [10, 11]. Furthermore, we propose the variation in needle length and the influence of thick clothes should be defined.

In future trials, we recommend the pressure applied to the ultrasound probe should be applied at the lowest and the highest pressure levels according to the specifications for each device.

Furthermore, the probe should have the same foot print as that of the specific EAI. This applies to both the EAIs available on the market at present as well as new brands or modifications of the presently available brands.

In our opinion, it is difficult to find a needle a length that would have no risk of intraosseous/periosteal injection and at the same time no risk of subcutaneous injection employing the present approach with the high pressure injection technique. Performing ultrasounds on individual patients could better estimate the risks in individual patients.

**Fig. 3:**
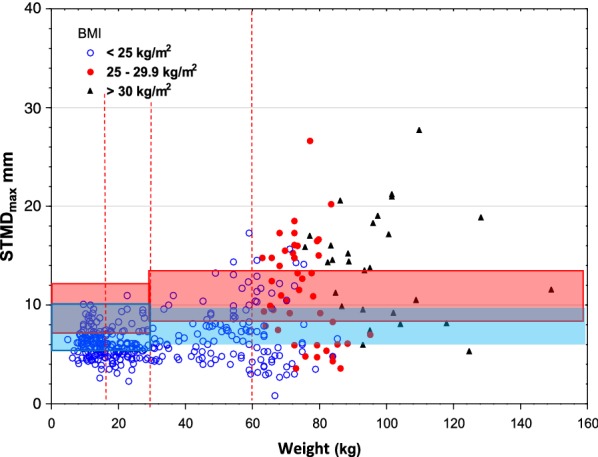
Skin to muscle distance, _max_, versus weight using Epipen^®^/EpipenJr^®^ EAIs. The legend to Fig. 1 explains the lines

**Fig. 4:**
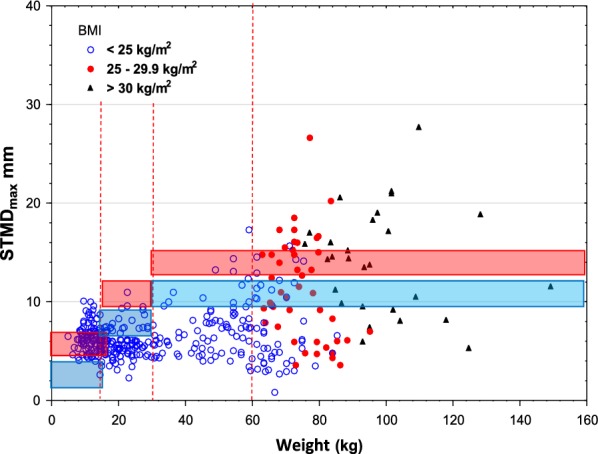
Skin to muscle distance_max_, versus weight using Auvi-Q^®^ EAIs. The legend to Fig. 1 explains the lines

**Fig. 5:**
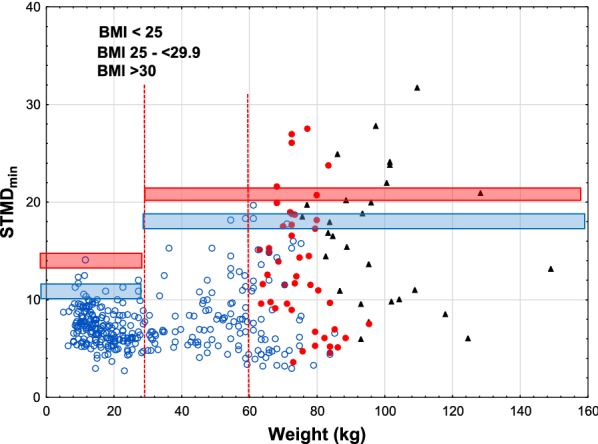
Skin to muscle distance_min_, versus weight using Emerade^®^ EAIs. The legend to Fig. 1 explains the lines

In the figures, we indicated BMI limits for adults. In adults, it seems that BMI does not add to the selection of obese patients for estimation of the risk of subcutaneous injection Figs. 3, 4 and 5.

Recently, Duong et al. presented data on BMI versus skin to muscle distance and skin to bone distance without considering the age dependant successive increase of BMI [12]. BMI-limits in childhood and adolescence must include evaluation using age dependent and puberty stage dependant  limits for BMI using z-scores. This is a complex task and will be investigated in the future.

Based on our ultrasound estimations of the skin to bone distance and skin to muscle distance, some EAIs, currently available in Europe and North America, do not likely deliver epinephrine intramuscularly in a significant number of patients. When wearing thick clothes, the risk of subcutaneous injection in overweight and especially obese patients is increased, and the risk of intraosseous/periosteal injection in young children using HPEAIs is reduced. In children weighing less than 15 kg the new Auvi-Q^®^ 0.1 mg EAI has no risk of intraosseous/periosteal injection but it has a 100% risk of subcutaneous injection wen injected through thick clothing. The only LPEAI, Emerade^®^, has a low risk of intraosseous injection, but a risk of subcutaneous injection in adult overweight/obese patients.

When developing and evaluating new EAIs and updating existing EAIs, it will be a challenge to balance the risk of subcutaneous injection and intraosseous/periosteal injection when considering the influence of thick winter clothing. There are some points that must be considered:

1. The variation of the length of the part of the needle exposed, i.e. the part of needle inserted in the thigh. The variation depends on the narrow or wide range of needle lengths approved in batches released for marketing. Every EAI of each brand can have a needle that is as long as the longest allowed by the batch release limits. The variation can be supervised and the range can be decreased by improved manufacturing processes. The needle length should be modified according to the pressures needed for needle release and injection.
2. The variation in pressure between EAIs of a specific brand allowed for batch release of that brand. We asked the manufacturers for this information who generously supplied this data [13]. No-one has investigated the influence of variation of pressure on the EAIs.
3. A third parameter is the variation of clothing. We now have shown the potential influences that clothing has on the delivery of epinephrine.
4. Children and adolescents grow and humans of all ages vary in weight and configuration. Therefore the risk of subcutaneous and intraosseous/periosteal injection will also vary individually from time to time. The only proper solution is to perform ultra-sound determination of skin to bone distance and skin to muscle distance every time an EAI is prescribed.
5. The choice between increased risk for subcutaneous and intraosseous/periosteal injection, between the Scylla of intraosseous/periosteal injection or the Carybdis of subcutaneous injection, is  depending on the other factors.

We believe that all of these papramerters must be taken into consideration in future studies.

## Conclusion

When injecting EAIs through thick clothes, the risk of subcutaneous injection is increased in all subjects, especially in overweight and obese patients. The risk of intraosseous/periosteal injection in young children using HPEAIs is reduced.

## Data Availability

Basic data are available in Excel format. Furthermore, basic information is available in the three original reports, references 2-4. The figures were produced by Statistical, using the Excel data.

## Abbreviations

EAI: Epinephrine auto-injector
HPEAI: High pressure EAI
LPEAI: Low pressure EAI
